# Role of Docosahexaenoic Acid Treatment in Improving Liver Histology in Pediatric Nonalcoholic Fatty Liver Disease

**DOI:** 10.1371/journal.pone.0088005

**Published:** 2014-02-04

**Authors:** Valerio Nobili, Guido Carpino, Anna Alisi, Rita De Vito, Antonio Franchitto, Gianfranco Alpini, Paolo Onori, Eugenio Gaudio

**Affiliations:** 1 Unit of Liver Research, Bambino Gesù Children's Hospital, Rome, Italy; 2 Department of Movement, Human and Health Sciences, University of Rome “Foro Italico”, Rome, Italy; 3 Unit of Pathology, Bambino Gesù Children's Hospital, Rome, Italy; 4 Department of Anatomical, Histological, Forensic Medicine and Orthopedics Sciences, Sapienza University of Rome, Rome, Italy; 5 Eleonora Lorillard Spencer-Cenci Foundation, Rome, Italy; 6 Department of Medicine, Scott & White and Texas A&M Health Science Center College of Medicine, Temple, Texas, United States of America; Univeristy of California Riverside, United States of America

## Abstract

**Introduction:**

Nonalcoholic fatty liver disease (NAFLD) is one of the most important causes of liver-related morbidity and mortality in children. Recently, we have reported the effects of docosahexaenoic acid (DHA), the major dietary long-chain polyunsaturated fatty acids, in children with NAFLD. DHA exerts a potent anti-inflammatory activity through the G protein-coupled receptor (GPR)120. Our aim was to investigate in pediatric NAFLD the mechanisms underlying the effects of DHA administration on histo-pathological aspects, GPR120 expression, hepatic progenitor cell activation and macrophage pool.

**Patients and Methods:**

20 children with untreated NAFLD were included. Children were treated with DHA for 18 months. Liver biopsies before and after the treatment were analyzed. Hepatic progenitor cell activation, macrophage pool and GPR120 expression were evaluated and correlated with clinical and histo-pathological parameters.

**Results:**

GPR120 was expressed by hepatocytes, liver macrophages, and hepatic progenitor cells. After DHA treatment, the following modifications were present: i) the improvement of histo-pathological parameters such as NAFLD activity score, ballooning, and steatosis; ii) the reduction of hepatic progenitor cell activation in correlation with histo-pathological parameters; iii) the reduction of the number of inflammatory macrophages; iv) the increase of GPR120 expression in hepatocytes; v) the reduction of serine-311-phosphorylated nuclear factor kappa B (NF-κB) nuclear translocation in hepatocytes and macrophages in correlation with serum inflammatory cytokines.

**Conclusions:**

DHA could modulate hepatic progenitor cell activation, hepatocyte survival and macrophage polarization through the interaction with GPR120 and NF-κB repression. In this scenario, the modulation of GPR120 exploits a novel crucial role in the regulation of the cell-to-cell cross-talk that drives inflammatory response, hepatic progenitor cell activation and hepatocyte survival.

## Introduction

Nonalcoholic fatty liver disease (NAFLD) is one of the most important causes of liver-related morbidity and mortality in children [1]. NAFLD includes a spectrum of diseases ranging from simple fatty liver to nonalcoholic steatohepatitis (NASH). NASH development in children is characterized by intricate interactions between resident and recruited cells that enable liver damage progression [1], [2]. Interestingly, we demonstrated that hepatic stem/progenitor cells (HPCs) may be involved in the response of the liver to oxidative stress in pediatric NAFLD, and their activation was correlated with fibrosis and NASH [3].

Recently, there has been growing interest in N-3 long-chain polyunsaturated fatty acids (LC-PUFA) supplementation as potential treatment for liver steatosis [4], [5]. The problems of adherence to lifestyle interventions to achieve sustainable weight loss especially in children, and side-effects with pharmacological agents make of dietary fish oil supplementation a simple and practical alternative therapy. Fish oil provides a convenient source of essential N-3 LC-PUFA with few side effects and may directly reduce hepatic lipogenesis and steatosis [5]–[7]. We have recently reported the short-term (6 months) and long-term (up to 24 months) effects of docosahexaenoic acid (DHA), the major dietary N-3 LC-PUFA, after 6, 12, 18 and 24 months of treatment with different concentrations (DHA 250 mg/day and DHA 500 mg/day) combined with diet and exercise [8], [9]. In these studies, algae DHA supplementation improved liver steatosis in children with NAFLD and was able to reduce the levels of serum ALT and triglycerides [8], [9].

DHA exerts a potent anti-inflammatory activity through the G protein-coupled receptor GPR120 [10]; dietary DHA suppresses hepatic markers of oxidative stress, inflammation and fibrosis [11]. However, its effects on histo-pathological features, as well as its cellular and molecular mechanisms in pediatric NAFLD remain to be established.

The aim of this study was to investigate the mechanisms underlying the effects of DHA administration in pediatric NAFLD (pNAFLD) on histo-pathological aspects, GPR120 expression, Macrophage (MΦ)/Kupffer cell(KC) polarization and HPC activation.

## Patients and Methods

### Ethics Statement

The study protocol conformed to the ethical guidelines of the 1975 Declaration of Helsinki. The study was approved by the local ethics committee (Bambino Gesù Children's Hospital IRCCS, Rome, Italy). A written informed consent was obtained from the next of kin, caretakers, or guardians on behalf of the children enrolled in this study.

### Patients, Anthropometrics and Laboratory Data

The study was performed on 20 patients from the randomised controlled clinical trial registered on http://www.clinicaltrials.gov (Trial identifier NCT00885313) conducted at the Liver Unit of the Bambino Gesù Pediatric Hospital (Rome, Italy). For the patients enrolment the entry criteria were: persistently elevated serum alanine transaminase (ALT ≥40 U/l), diffusely hyperechogenic liver at ultrasonography and liver biopsy consistent with NAFLD. Furthermore, patients with secondary causes of steatosis, including alcohol abuse (≥140 g/week), total parenteral nutrition, drugs, hepatitis A, B, C, cytomegalovirus, Epstein-Barr virus infections, autoimmune liver disease, metabolic liver diseases, Wilson’s disease, and alpha-1-antitrypsin deficiency were excluded from the trial. The children included in our analyses showed clinical and pathological features resembling those seen in our general pediatric population with NAFLD [12].

These patients were diagnosed with NAFLD through liver biopsy recommended because of persistently (>6 months) elevated ALT levels and the presence of an echogenic texture of the liver on ultrasonography. Furthermore, the patients received no dietary or other therapeutic treatment regimens before diagnosis.

The clinical indication for biopsy was either to assess the presence of NASH and the degree of fibrosis or to diagnose other likely independent or competing liver diseases. Liver biopsy was performed after an overnight fast by using an automatic core biopsy 18-gauge needle under general anesthesia and ultrasound guidance. Two biopsies passing within different liver segments were performed for each subject.

Patients were treated with DHA 250 mg/day (39% DHA oil obtained from Schyzochitrium, Martek Biosciences Corporation, Columbia, MD, USA). Liver biopsy was performed after 18 months of treatment.

Liver specimens from 6 lean, non-diabetic children (boys, 4; girls, 2; median age: 13 years, range, 12–16 years) without liver disease were used as controls. Control liver fragments were obtained from patients who underwent laparotomy or laparoscopic procedures (for cholecystectomy), from liver donors (orthotopic liver transplantation) or incidental “normal” liver biopsies (children exhibiting persistent/intermittent elevations of liver enzymes for >6 months).

Weight, height, and body mass index (BMI) were measured [12]. Alanine and aspartate aminotransferase, gamma-glutamyl-transpeptidase, total triglycerides and total cholesterol were evaluated using standard laboratory methods. Insulin was measured using a radio-immunoassay (Myria Technogenetics, Milan, Italy). Glucose and insulin were measured at 0, 30, 60, 90 and 120 minutes of an oral glucose tolerance test performed with 1.75 grams of glucose per kilogram of body weight (up to 75 g). The degree of insulin resistance and sensitivity was determined by the homeostatic model assessment insulin resistance (HOMA-IR), and the insulin sensitivity index (ISI) derived from an oral glucose tolerance test (OGGT), respectively. Data at baseline and after 18 month of treatment were acquired.

### Liver Ultrasonography

Liver ultrasonography was performed by an experienced radiologist using an Acuson Sequoia C512 scanner equipped with a 15L8 transducer (Universal Diagnostic Solutions, Oceanside, CA). Steatosis has been graded by a validated score [13]. Absent steatosis (grade 0) was defined as normal liver echo-texture; mild steatosis (grade 1) as slight and diffuse increase in fine parenchymal echoes with normal visualization of diaphragm and portal vein borders; moderate steatosis (grade 2) as moderate and diffuse increase in fine echoes with slightly impaired visualization of diaphragm and portal vein borders; and severe steatosis (grade 3) as fine echoes with poor or no visualization of diaphragm, portal vein borders and posterior portion of the right lobe.

### Histopathology, Immunohistochemistry (IHC) and Immunofluorescence (IF)

Steatosis, inflammation, hepatocyte ballooning and fibrosis were scored using the NAFLD Clinical Research Network (CNR) criteria [14]. Steatosis, lobular inflammation, and hepatocyte ballooning were combined to obtain the NAFLD activity score (NAS). A microscopic diagnosis based on overall injury pattern (steatosis, hepatocyte ballooning, inflammation) as well as the presence of additional lesions (e.g. zonality of lesions, portal inflammation and fibrosis) has been assigned to each case [15]. Accordingly, biopsies were classified into: not steatohepatitis (not-SH), definite steatohepatitis (definite-SH), borderline zone 1 pattern or borderline zone 3 pattern subcategories [15]. PNHS, a more adequate score for pNAFLD [16], was also calculated. Histological analysis was performed by a single pathologist blinded to clinical and laboratory data.

For IHC and IF, sections were incubated overnight at 4°C with primary antibodies against cytokeratin (CK)7 (Dako, mouse monoclonal, code: M7018, dilution: 1∶100), EpCAM (Dako, mouse monoclonal, code: M3525, dilution: 1∶100), CD68 (Dako, mouse monoclonal, code: M0876, dilution: 1∶100), GPR120 (Sigma-Aldrich, rabbit polyclonal, code: HPA-042563, dilution: 1∶30), and serine-311-phosphorylated NF-κB (p-65NF-κB) purchased from Santa Cruz Biotechnology (goat polyclonal, code: sc-33039-R, dilution 1∶50). For IHC, samples were than incubated for 20 minutes at room temperature with secondary biotinylated antibody and, successively, with streptavidin-Horse radish peroxidase (LSAB+, Dako, code K0690). Diaminobenzidine (Dako, code K3468) was used as the substrate and the sections were counterstained with hematoxylin. For double IF staining, non-specific protein binding was blocked with 5% normal goat serum. After the incubation with primary antibodies, specimens were washed and incubated for 1 h with labeled isotype-specific secondary antibodies (anti-mouse AlexaFluor-488 and anti-rabbit Alexafluor-564, Invitrogen) and counterstained with 4,6-diamidino-2-phenylindole (DAPI) for visualization of cell nuclei. For all immunoreactions, negative controls (the primary antibody was replaced with pre-immune serum) were also included [17].

Sections were examined with a Leica Microsystems DM 4500 B Microscopy (Weltzlar, Germany) equipped with a Jenoptik Prog Res C10 Plus Videocam (Jena, Germany). Only biopsies containing at least 5 portal spaces were considered [18].

The ductular reaction (DR) extension was evaluated on liver section stained for CK7. Slides were scanned and processed by a digital scanner (Aperio Scanscope CS System, Aperio Technologies, Inc). The virtual slide have been visualized and processed by the software included in the Aperio Scanscope CS System (ImageScope). An image analysis algorithm, that automatically analyzes, the digital slides has been used to quantify the area occupied by CK7 positive cells. The algorithm was applied on the entire sections. The extent of DR was expressed as the percentage of the parenchymal area occupied by reactive ductules [19]. Solitary CK7-positive HPCs or those in small clumps that were localized in the parenchyma or at the portal interface were included in these counts because they should be considered as a histological sectioning of bile/reactive ductules through a transverse plane without any unique immunohistochemical markers to distinguish them from cells within bile/reactive ductules [19] . Cholangiocytes lining the interlobular bile ducts were excluded from the counts.

The activation of HPC compartment was evaluated by counting the number of EpCAM-positive cells within the bile/reactive ductules and expressed as number of positive cells per high power field (HPF: at 40×) [20].

Intermediate hepatocytes (IHs) were defined as cells with sizes between those of hepatocytes and HPCs (<40 but >6 µm in diameter), with faint CK-7 immunoreactivity in the cytoplasm and reinforcement at the plasma membrane7. The presence of IHs was scored as reported elsewhere: 0 = no IH, 1 = single occasional IHs and 2 = clusters of His [21].

GPR120 expression by EpCAM-positive HPCs or CD68-positive macrophages was evaluated in serial sections and by double IF. The number of positive macrophages per HPF was calculated. GPR120 expression by hepatocytes and HPCs was semi-quantitatively scored [22]. The nuclear expression of p-65NF-κB was calculated counting the number of MΦ/KC or hepatocytes with positive nuclei. Data were expressed as a percentage of positive cells. At least 30 lobular fields at 40× magnification were analyzed for each section [3].

### Cytokine Assay

Serum levels of pro-inflammatory cytokines were determined using cytokine-specific ELISA assays. Concentrations were evaluated by manufacturing protocols from RayBiotech Inc (Norcross GA, USA) for IL-1β and IL-6 and from Immundiagnostik (AG, Bensheim, Germany) for tumour necrosis factor (TNF)-α.

### Statistical Methods

Data are indicated as mean±standard deviation (SD). The Student t test or Mann–Whitney U test was used to determine differences between groups for normally or not normally distributed data, respectively. To evaluate the modification of variables after DHA treatment, the paired-samples T Test was applied. The Pearson correlation coefficient or the Spearman nonparametric correlation were used. A *p*-value of <0.05 was considered statistically significant. Analyses were performed using SPSS software.

## Results

### Clinical and Histological Data

We evaluated 20 patients enrolled in the aforementioned trial before and after DHA-250 treatment (18 months) [8]. With regard to anthropometric and laboratory data (**Table 1**), total triglycerides, ALT, AST and ISI were significantly reduced after DHA treatment while no differences were found in BMI, GGT, total cholesterol, basal glycemia and insulinemia and HOMA-IR. Finally, the steatosis grade assessed by liver ultrasonography was strongly ameliorated after DHA treatment (p<0.001). Serum levels of pro-inflammatory cytokines (IL-1β, IL-6 and TNF-α) were significantly reduced after DHA treatment.

**Table 1:** Anthropometric and laboratory parameters of the study-population.

	Before DHATreatment	After DHAtreatment	Paired-samplesT Test (p-value)
**Sex (M/F)**	11/9	11/9	–
**Age (years)**	10.1±2.0	12.3±2.0	<0.001*
**Weight (kg)**	57.1±9.9	59.8±9.9	<0.001*
**Height (cm)**	151±17	165±16	<0.001*
**BMI (kg/m2)**	26.1±2.5	25.63±2.2	0.072
**Cholesterol (mg/dL)**	172.1±11.2	169.6±12.9	0.729
**Triglycerides (mg/dL)**	91.9±30.1	78.8±17.7	0.048*
**ALT (U/L)**	65.4±21.3	38.9±6.2	0.001*
**AST (U/L)**	50.0±15.7	35.4±5.8	0.007*
**GGT (U/L)**	21.3±5.6	19.1±3.1	0.204
**Glucose (mg/dL)**	84.9±6.7	86.6±3.7	0.328
**Insulin (µU/mL)**	14.7±8.1	12.0±4.8	0.057
**HOMA-IR**	3.1±1.8	2.5±1.0	0.089
**ISI**	3.0±0.5	3.4±0.5	0.038*
**TNF-alpha (pg/mL)**	19.85±3.32	5.89±2.09	<0.001*
**IL-6 (pg/mL)**	12.55±1.32	5.85±2.23	<0.001*
**IL-1B (pg/mL)**	25.10±1.46	8.87±2.86	<0.001*
**US**	2.6±0.5	1.3±0.6	<0.001*

Data are reported as Means ± SD * = p<0.05.

Before the treatment, the biopsies were classified in accordance to the NASH CNR criteria [14], [23], into 4 distinct groups (**Supplementary** **Figure 1**): not-SH (N = 3), definite-SH or NASH (N = 12), borderline zone1 pattern (N = 1), and borderline zone3 pattern (N = 4). NAS scores ranged from 1 to 7. PNHS scores ranged from 1.60 to 100. Fibrosis of some degree was seen in all biopsy samples: stage 1c in 9 samples, stage 2 in 10, and stage 3 in 1. Control biopsy samples had normal histological features.

**Figure 1:**
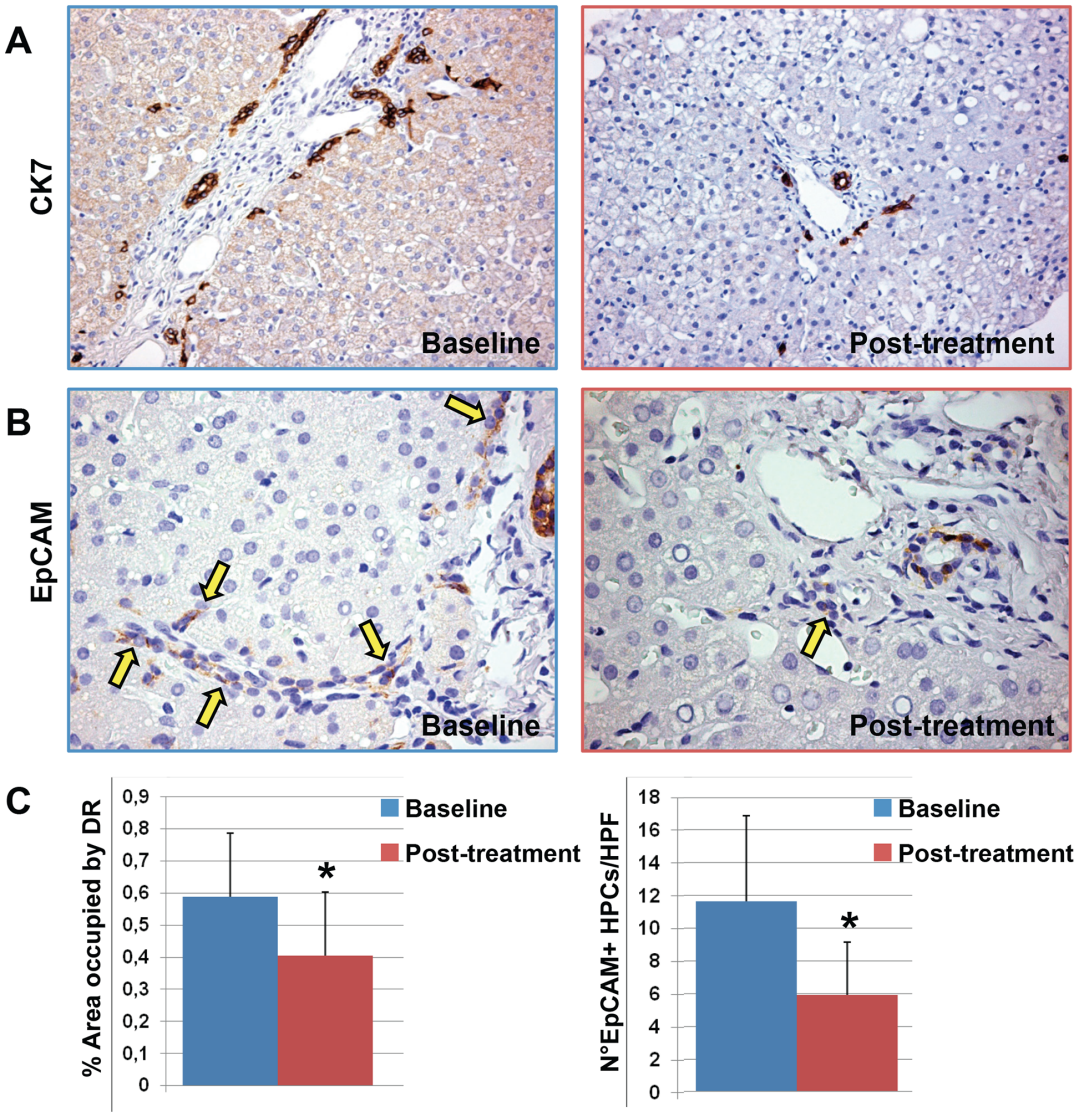
Immunohistochemistry for Cytokeratin(CK) 7 and EpCAM in liver biopsies of pediatric NAFLD patients. A) at the beasline, pediatric NAFLD biopsies were characterized by a prominent expansion of hepatic progenitor cell (HPC) pool and the presence of reactive ductules at the periphery of portal spaces. After DHA treatment, a minimal involvement of the HPC compartment was present. Original magnification 20×. B) EpCAM-positive HPCs (arrows) were significant reduced by DHA treatment in comparison with the biopsies at the baseline. Original magnification 40×. C) Histograms show the reduction of HPC expansion (ductular reaction extension) and activation (number of EpCAM+ cells) after DHA treatment. Data are shown as Means ± Standard Deviation. * = p<0.05.

After the treatment with DHA, the classification of biopsies indicated a decrease of definite-SH or NASH diagnosis (N = 3) and an increase of not-SH diagnosis (N = 15).

Moreover, NAS (t = 5.69; p<0.001), PNHS score (t = 2.61; p<0.05), steatosis (t = 4.86; p<0.001), ballooning (t = 5.34; p<0.001) and lobular inflammation (t = 2.35; p<0.05) were significantly reduced (**Table 2**), while no differences were found in fibrosis.

**Table 2:** Histo-pathologic evaluation of the study-population.

	Before DHATreatment	After DHA treatment	Paired-samplesT Test (p-value)
**Steatosis**	1.70±1.08	0.50±0.61	<0.001
**Ballooning**	0.85±0.67	0.25±0.44	<0.001
**Lobular inflammation**	1.15±0.59	0.85±0.37	<0.05
**NAS**	3.70±1.78	1.60±1.05	<0.01
**PNHS**	71.31±40.84	5.8±8.3	<0.05
**Fibrosis**	1.60±0.60	1.45±0.76	= 0.481

Data are reported as Means ± SD.

### Effect of DHA Treatment on HPCs in pNAFLD Biopsy Samples

Before treatment, the pNAFLD biopsy samples showed an expanded DR (0.59±0.20) compared with those of normal livers (0.21±0.07, p<0.01). After DHA treatment, the DR expansion was reduced when a test for paired samples was applied (t = 3.521, p<0.01; **Figure 1A** **/C**). Moreover, DR was correlated with NAS (r = 0.458; p<0.05) and PNHS scores (r = 0.478; p<0.05) and with ballooning (r = 0.526; p<0.05) but not with fibrosis, hepatic steatosis or lobular inflammation.

Then, we evaluated the number of EpCAM-positive cells per HPF (**Figure 1B** **/C**). EpCAM is considered a specific marker of activated HPCs and several lines of evidence indicated that EpCAM expression is tightly regulated and only occurs in case of a temporary need for proliferation and is immediately down-regulated upon terminal differentiation [24], [25]. Before treatment, pNAFLD biopsy samples showed an expanded number of EpCAM-positive HPCs (11.64±5.24) compared with those of normal livers (3.54±1.15; p<0.01). After DHA treatment, the number of EpCAM-positive HPCs was statistically reduced when a test for paired samples was applied (t = 4.102, p<0.01). Consistently, the number of EpCAM-positive HPCs was strictly correlated with the extension of DR (r = 0.625; p<0.01). Moreover, the number of EpCAM-positive HPCs was strictly correlated with NAS (r = 0.606; p<0.01) and PNHS scores (r = 0.595; p<0.01), with hepatic steatosis (r = 0.499; p<0.05) and ballooning (r = 0.683; p = 0.01) but not with fibrosis or lobular inflammation. Taking in account the diagnostic categories, the number of EpCAM-positive HPCs was significantly lower in not-SH in comparison with biopsies with definite-SH diagnosis (t = 2.704; p<0.02).

As regard clinical parameters, DR extension and the number of EpCAM-positive HPCs were correlated with ALT (r = 0.467; p<0.05 and r = 0.657; p<0.01, respectively) and with the steatosis grade assessed by liver ultrasonography (r = 0.490; p<0.05 and r = 0.632; p<0.01, respectively).

### GPR120 Expression and its Correlation with Histo-pathological Parameters

**Figure 2:**
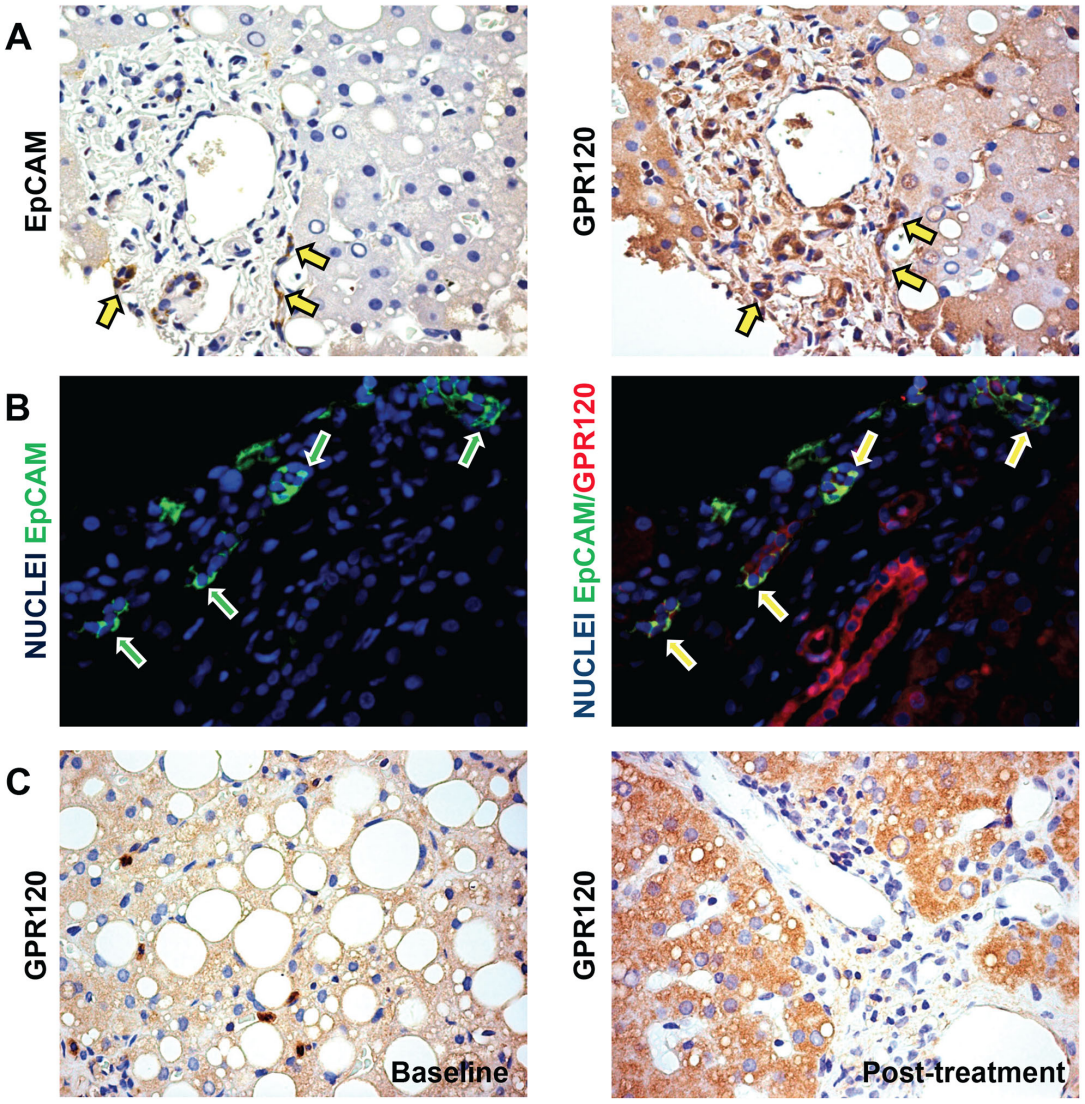
GPR120 expression by HPC and Hepatocytes in pediatric NAFLD patients. A) Immunohistochemistry for EpCAM and GPR120 in serial sections of pediatric NAFLD biopsies. CK-7-positive HPCs highly express GPR120 (arrows). B) Double immunofluorescence for EpCAM (green) and GPR120 (red) in NAFLD biopsies. Nuclei are shown in blue. Numerous EpCAM-positive progenitor cells co-express GPR120 (yellow cells: yellow arrows) confirming the findings obtained through immunohistochemistry in serial sections. C) Immunohistochemistry for GPR120 in sections of pediatric NAFLD biopsies before and after DHA treatment. A significant increase of GPR120-positive hepatocytes was clearly seen after DHA treatment. Original magnification: 40×.

In liver biopsies, GPR120 was expressed by hepatocytes, cholangiocytes, HPCs and MΦ/KC pool as seen both in IHC sections and through double IF with specific cell markers (**Figures 2** **,** **3**).

**Figure 3:**
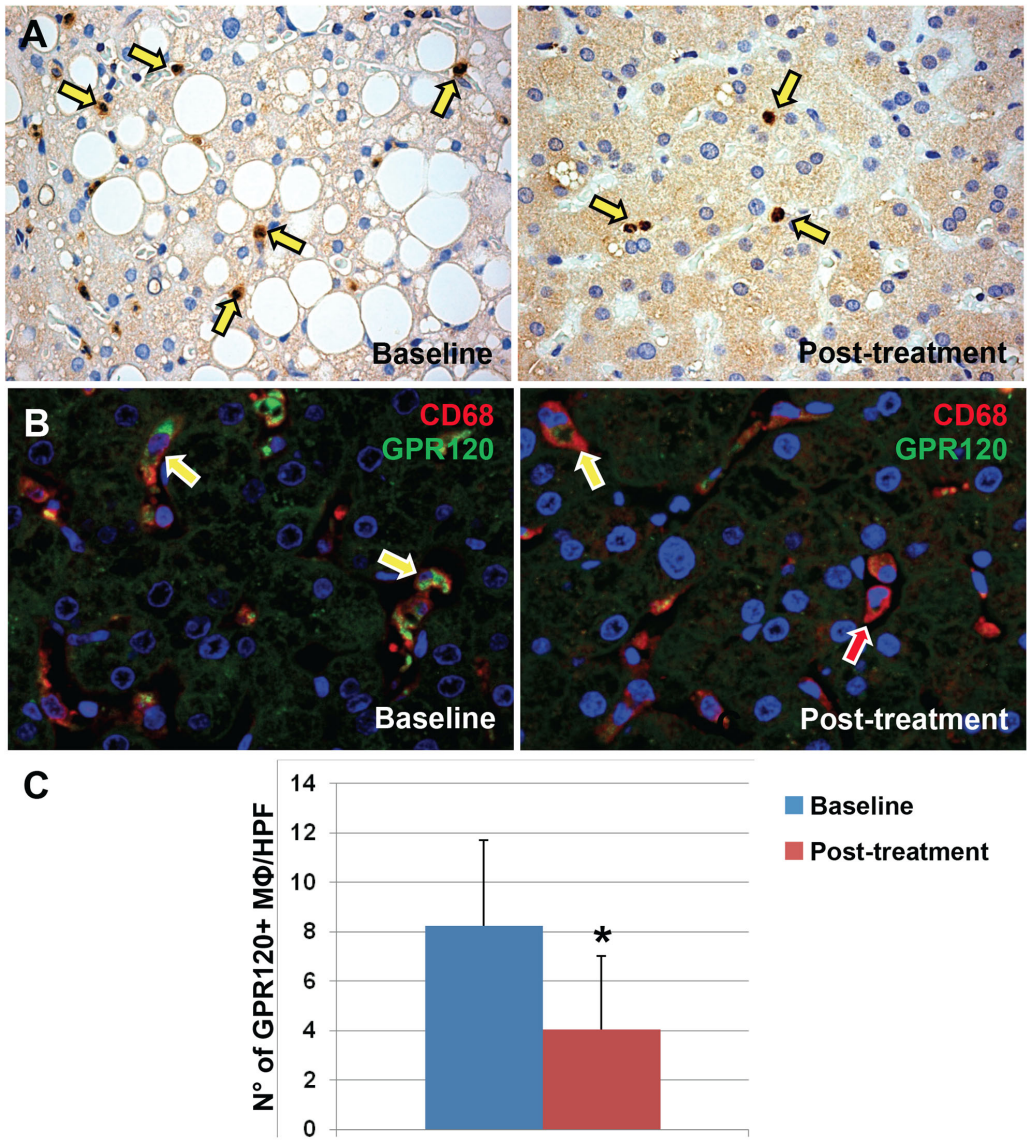
GPR120 expressions by Macrophages in pediatric NAFLD patients. A) Immunohistochemistry for GPR120 in sections of pediatric NAFLD biopsies. B) Double immunofluorescence for CD68 (red) and GPR120 (green) in NAFLD biopsies. Nuclei are shown in blue. CD68-positive macrophages express GPR120 (yellow arrows). Original magnification: 40×. C) The histogram shows the number of GPR120-positive macrophages per high power field (HPF) at the baseline and after DHA treatment. A significant reduction of GPR120-positive macrophages was seen after DHA treatment. * = p<0.05.

GPR120 was also expressed by HPCs as shown in serial sections and in double IF staining (**Figure 2A** **/B**). No significant differences were found comparing pNAFLD biopsies before and after DHA treatment. However, considering all pNAFLD biopsy samples, the overall GPR120 expression by HPCs was inversely correlated with the presence of intermediate hepatocytes (r = −0.496, *p*<0.05), NAS score (r = −0.501, *p*<0.05) and steatosis (r = −0.575, *p* = 0.01).

With regard GPR120 expression in hepatocytes (**Figure 2C**), a higher expression of GPR120 was observable (2.33±0.88) in biopsies after DHA treatment when compared with pre-treatment biopsies (1.58±1.08; t = −3.00; p<0.05). Moreover, when the diagnostic categories were taken in account, the number of GPR120 expression in hepatocytes was significantly higher in not-SH in comparison with biopsies with definite-SH diagnosis (t = −2.223; p<0.05). In addition, the expression of GPR120 by hepatocytes was correlated with basal glycemia (r = −0.505, *p*<0.05).

The number of GPR120-positive MΦ/KC has been evaluated in IHC sections and by double IF with the specific marker, CD68 (**Figure 3**). CD68 clearly and specifically recognized MΦ/KC in liver sinusoids but was consistently negative in other liver cells.

Evaluation of GPR120 expression showed that the number of MΦ/KC expressing GPR120 per HPF was increased in pNAFLD patients (8.25±3.47) in comparison with normal livers (2.83±1.47, p<0.01). Moreover, a significant reduction (**Figure 3**) in the number of GPR120-positive MΦ/KC (4.08±2.97) within the liver (t = 4.20; p<0.01) was observable after the treatment with DHA.

**Figure 4:**
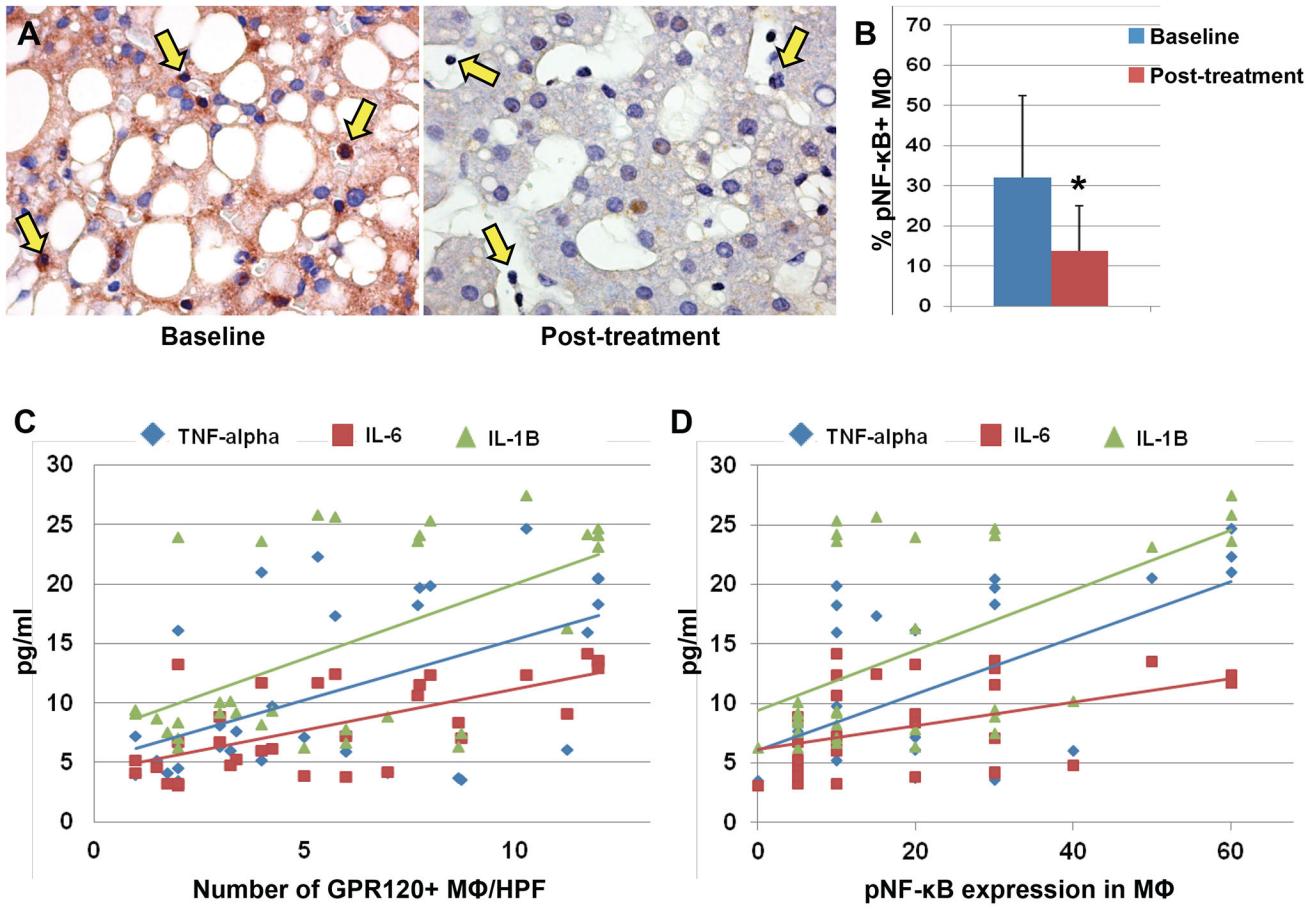
Nuclear expression of phosphorylated (p) NF-κB by Macrophages in pediatric NAFLD patients. A) Immunohistochemistry for serine-311-phosphorylated NF-κB (pNF-κB) in pediatric NAFLD biopsies before and after DHA treatment. In parallel with GPR120 expression, the DHA treatment determined a reduction of pNF-κB expression and its nuclear translocation in macrophages (A, yellow arrows). Original magnification: 40×. B) Histogram shows the significant reduction of pNF-κB nuclear translocation in macrophages after DHA treatment. Data are shown as Means ± Standard Deviation. * = p<0.05. C–D) The number of GPR120-positive macrophages (C) and the pNF-κB nuclear expression in macrophages. (D) were correlated with serum level of inflammatory cytokines.

Interestingly, the number of GPR120-positive MΦ/KC per HPF was directly correlated with serum levels of pro-inflammatory cytokines (TNF-alpha r = 0.519, p<0.01; IL-6: r = 0.670, p<0.001 and IL-1B: r = 0.569, p<0.04: **Figure 4C**). In addition, the number of GPR120-positive MΦ/KC per HPF was inversely correlated with the presence of intermediate hepatocytes (r = −0.512, *p*<0.05).

### Phosphorylated(p) NF-κB Expression in Macrophages and Hepatocytes

As phosphorylation of NF-κB at serine 311 is important for its TNF-dependent binding to p300 coactivator and recruitment to target promoters after nuclear translocation, we evaluated p-NF-κB expression in our patients. We found that p*-*NF-κB was expressed by hepatocytes and MΦ/KCs at cytoplasmic and nuclear levels (**Figures 4A** **–** **5A**). The percentage of cells, which displayed a nuclear translocation of p-NF-κB, has been determined. The percentages of hepatocytes and MΦ/KCs expressing p-NF-κB was reduced in pNAFLD patients after the treatment with DHA (**Figure 4B** **–** **5B**) when the paired-samples T Test was applied (t = 2.21 and t = 2.94, respectively p<0.05). In addition, the percentage of p-NF-κB-positive hepatocytes was directly correlated with NAS, ductular reaction and activation of HPCs (p<0.05), whereas the macrophage expression of p-NF-κB was correlated with NAS and ductular reaction extension (p<0.05). Interestingly, the nuclear expression of p-NF-κB in macrophages was strongly correlated with serum levels of pro-inflammatory cytokines (TNF-α: r = 0.585; p<0.001; IL-6: r = 0.467, p<0.01; IL-1B: r = 0.547, p<0.001, **Figure 4D**). Finally, the number of GPR120-positive macrophages was correlated with the expression of p-NF-κB (r = 0.384; p<0.05).

**Figure 5:**
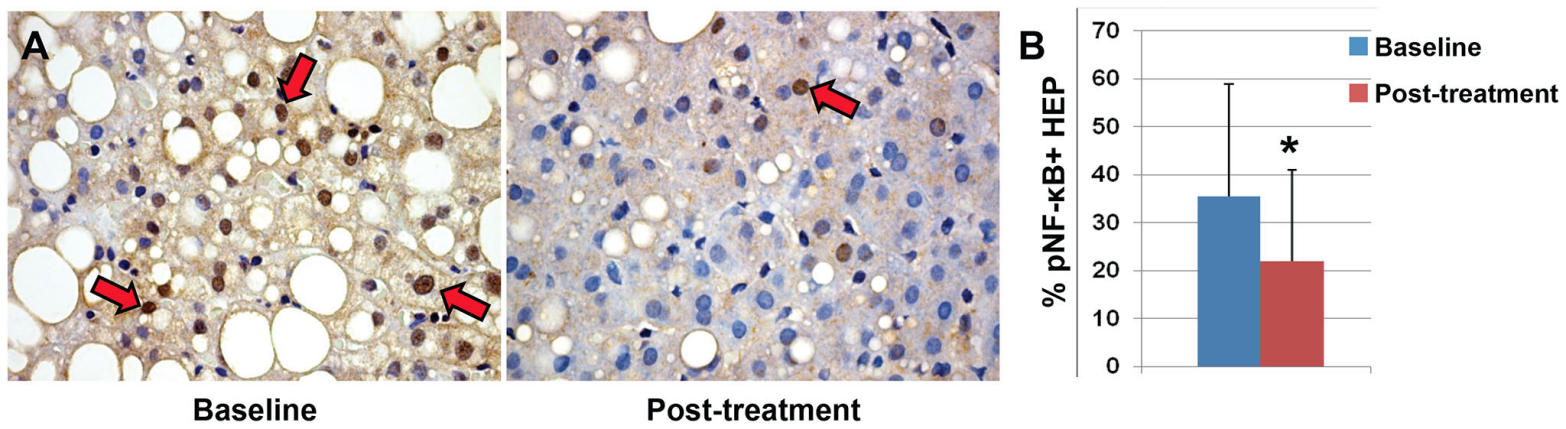
Nuclear expression of phosphorylated (p) NF-κB by hepatocytes in pediatric NAFLD patients. A) Immunohistochemistry for pNF-κB in pediatric NAFLD biopsies demonstrates the reduction of pNF-κB in hepatocyte nuclei (red arrows) after DHA treatment. B) DHA treatment determined a significant reduction of pNF-κB expression and its nuclear translocation in hepatocytes. Data are shown as Means ± Standard Deviation. * = p<0.05.

## Discussion

This is the first follow-up study demonstrating the liver specific effects of 18-months treatment with DHA in pNAFLD which (i) improves hepatic steatosis, ballooning, inflammation NAS and PNHS scores but was ineffective on fibrosis; (ii) causes a remarkable reduction of DR and HPC activation strongly associated with NAS and PNHS scores; (iii) induces a reduction of GPR120-positive inflammatory MΦ/KCs and an increase of GPR120-positive hepatocytes; and (iv) downregulates nuclear translocation rate of phosphorylated form of p65NF-κB in hepatocytes and liver macrophages.

In a recently completed randomized clinical trial, we found that a 24-months supplementation with DHA was able to determine a decrease in liver fat [9]. Clinically speaking, in pediatric cohort of this study, decreases at 18-months in total triglycerides, ALT, AST and ISI were observed in DHA-supplemented children but their estimates were imprecise and larger sample sizes are needed to adequately estimate these effects. In the present study, DHA treatment ameliorated hepatocyte steatosis, ballooning, NAS and PNHS scores. Taking in account the diagnostic category, no treated patient had a progression of the histo-pathological damaging and a worse diagnosis at the biopsy after the 18-months therapy. Moreover, 13/17 patients, which were classified as definite SH or borderline before the treatment, presented a more positive diagnosis after the treatment.

A limitation of the present study could be represented by the lack of biopsies from a placebo group. In the completed randomized clinical trial, clinical, laboratory and liver ultrasound data were used to discriminate effects between DHA treated and placebo groups [26]. Indeed, considering that patients were children, the placebo group did not receive liver biopsy at 18 months.

LC-PUFAs exert their actions by the modulation of GPR120 [10], [27]. Here, we showed that GPR120 was expressed by several cell types in human liver as MΦs/KCs within sinusoids, hepatocytes and HPCs. Accordingly, the clinical effects and the histo-pathological changes after DHA treatment in pNAFLD patients could be determined through the DHA action on all these different cells.

GPR120 has been indicated as a lipid-sensing receptor highly expressed in proinflammatory macrophages [10]. By signaling through GPR120, DHA mediates potent anti-inflammatory effects by inhibiting both Toll-Like Receptor (TLR) and Tumor Necorsis Factor(TNF)-α signaling pathways [10], [27]. Moreover, GPR120 seems to have a key role in counter-inflammation representing a feedback signal that blocks NF-κB phosphorylation [27], [28]. Our results were in accordance with this scenario. In pNAFLD, the number of GPR120-positive (inflammatory) MΦ/KCs was increased in comparison with normal. Interestingly, DHA treatment induced a significant reduction of GPR120-positive MΦ/KC pool that matched with a strong decrease of macrophages expressing serine 311 phosphorylated form of NF-κB at nuclear level. These results suggested that, in pNAFLD, DHA treatment was able to reduce the inflammatory macrophage pool and induce an anti-inflammatory macrophage polarization. Two distinct modes of macrophage activation were proposed to differentiate between M1 and M2 macrophages [29]. M1-macrophages produce pro-inflammatory cytokines such as Interleukin (IL)-1β, IL-6, IL- 8, IL-12, and TNF-α. Macrophage can be polarized toward alternative activation phenotype (M2) which has a role in promoting wound healing through the releasing of anti-inflammatory cytokines and the promotion of a efficient clearance of apoptotic cells [30].

In keeping, GPR120 macrophage expression was strongly correlated with the serum levels of pro-inflammatory cytokines (TNF-α, IL-6 and IL-1β). These data seem to confirm that, in pNAFLD, GPR120-positive macrophages within the liver are responsible of pro-inflammatory cytokine production and the treatment with DHA could switch the macrophage polarization via the modulation of NF-κB pathway.

DHA treatment caused a decrease of DR and HPC activation that was strongly correlated with the improvement of clinical and imaging parameters, hepatocyte steatosis and ballooning, NAS and PNHS. Noteworthy, the activation of HPC compartment was higher in definite steatohepatitis compared with not steatohepatitis biopsies. Taken together, these results highlight the pivotal role of HPC activation in pNAFLD and its potential impact on the progression towards NASH.

Recently, we demonstrated that HPC expansion, especially in children with NASH, is associated with the degree of liver injury, hepatocyte apoptosis, and cell-cycle arrest [3]. In general, the local microenvironment has a key role in achieving a defined progenitor specification in response to diverse injuries [20], [31]. Recently, macrophages have been indicated as a key component of HPC niche [31]; during hepatocyte regeneration, an inefficient efferocytosis of hepatocyte debris could be able to induced Wnt3a expression by macrophages; this resulted in canonical Wnt signaling in nearby HPCs, thus promoting their specification to hepatocytes [31]. Interestingly, in the present study, the presence of intermediate hepatocytes, a sign of HPC differentiation towards hepatocyte fate, was correlated with the number of GPR120-positive macrophages. In this context, DHA could exert a dual effect on HPC niche acting directly on HPCs by GPR120 modulation or indirectly on macrophage polarization [20], [32].

Our results indicated that GPR120 hepatocyte expression was highly enhanced by treatment with DHA and was higher in not steatohepatitis in comparison with definite SH biopsies. Parallel, DHA determined a reduction of pNF-kB in hepatocyte nuclei. The activation of TLR4 signaling in hepatocytes, accompanied with the relocation of NF-κB into the nucleus, was proven to play an important role during the initiation of NAFLD [33], [34]. Our data showed that the nuclear expression of pNF-κB in hepatocytes was correlated with the serum levels of pro-inflammatory cytokines, thus suggesting that NF-kB activation and its modulation in hepatocytes could have a role in liver and systemic inflammatory status.

In conclusion, our results highlight the anti-inflammatory effects of DHA administration in pNAFLD and the liver-resident cells which could be stimulated through the interaction with GPR120-mediated NF-κB pathway. Furthermore, in this scenario, LC-PUFA-mediated modulation of GPR120 exploits a novel crucial role in the regulation of the cell-to-cell cross-talk that controls lipid homeostasis, insulin sensitivity, danger recognition and immune tolerance response.

## Supporting Information

Figure S1 **Histopathological aspects of pediatric NAFLD biopsies.** A) The different diagnostic categories in which biopsies were characterized were showed: definite steatohepatitis (Nash: NAS Score ≥5); Nash Borderline (NAS Score = 3–4); Not-steatohepatitis (Not Nash: NAS score = 1–2); B) liver biopsies at baseline and after DHA treatment. Steatosis and ballooning were significantly reduced after the DHA treatment. Hematoxylin-Eosin. Original Magnification: 10X. C) Histo-pathological evaluation: NAS, steatosis, ballooning, and lobular inflammation were significantly reduced after the DHA treatment while no differences were found in fibrosis. * = p<0.05.
(TIF)
