# Supplementary figures and images for: Role of Docosahexaenoic Acid Treatment in Improving Liver Histology in Pediatric Nonalcoholic Fatty Liver Disease
Source: PLoS One. 2014 Feb 4;9(2):e88005. doi: 10.1371/journal.pone.0088005 (PMC3913708; doi:10.1371/journal.pone.0088005)

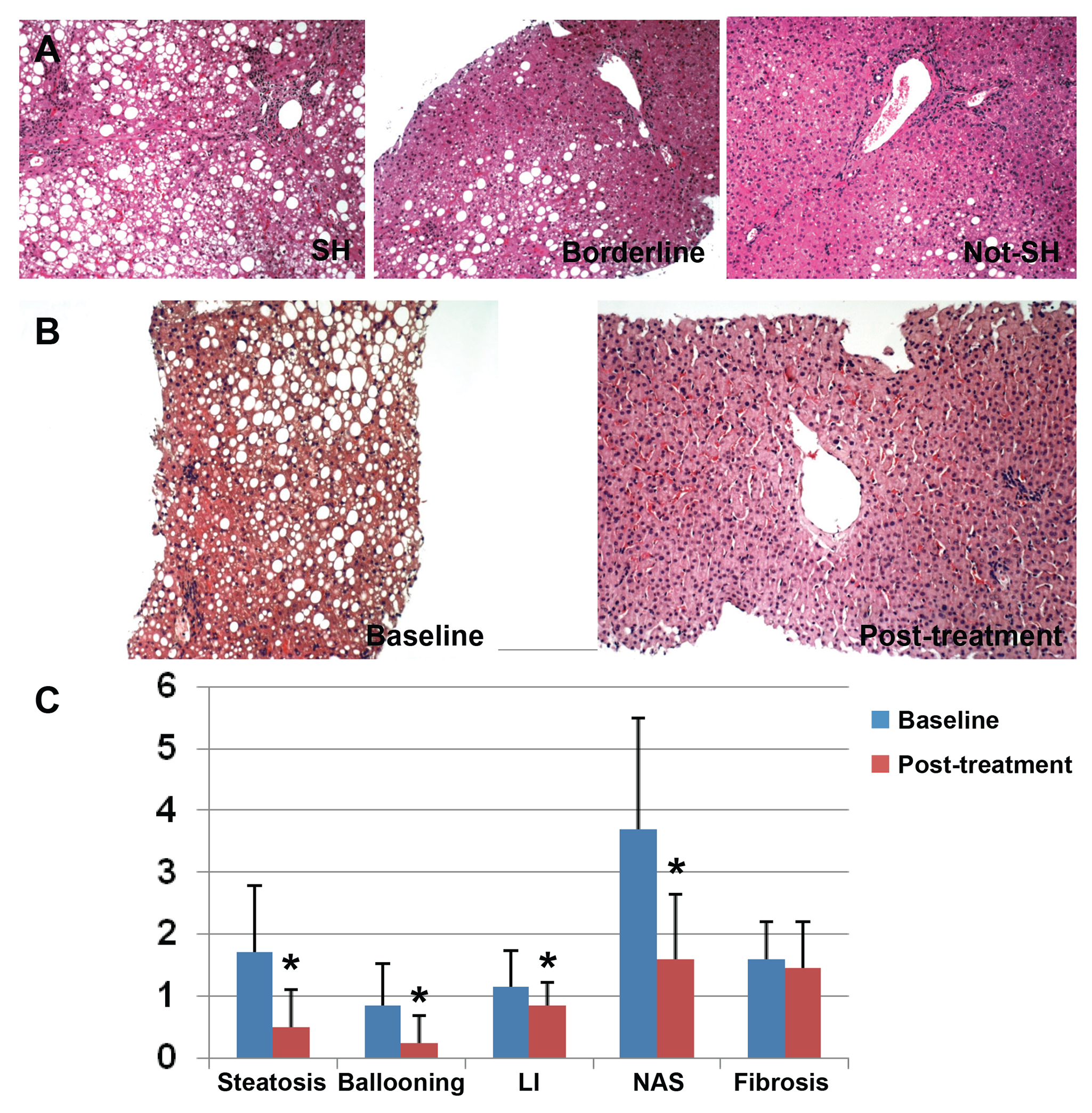

Supplement: Figure S1 — Histopathological aspects of pediatric NAFLD biopsies. A) The different diagnostic categories in which biopsies were characterized were showed: definite steatohepatitis (Nash: NAS Score ≥5); Nash Borderline (NAS Score = 3–4); Not-steatohepatitis (Not Nash: NAS score = 1–2); B) liver biopsies at baseline and after DHA treatment. Steatosis and ballooning were significantly reduced after the DHA treatment. Hematoxylin-Eosin. Original Magnification: 10X. C) Histo-pathological evaluation: NAS, steatosis, ballooning, and lobular inflammation were significantly reduced after the DHA treatment while no differences were found in fibrosis. * = p<0.05. (TIF) [file pone.0088005.s001.tif]
